# Higher adverse childhood experiences interference with targeted early intervention to reduce persistence of adult subacute pain: a feasibility open trial

**DOI:** 10.3389/fpsyg.2023.1270598

**Published:** 2023-11-23

**Authors:** Joyce McSwan, Peta Stapleton, Catherine E. Panwar

**Affiliations:** ^1^PainWise Pty Ltd., Varsity Lakes, QLD, Australia; ^2^School of Psychology, Bond University, Varsity Lakes, QLD, Australia; ^3^Panwar Health Pty Ltd., Wamberal, NSW, Australia

**Keywords:** subacute pain, pain management, adverse childhood experiences, early intervention program, primary healthcare

## Abstract

**Introduction:**

The feasibility of an Early Subacute Pain Intervention Program was assessed for improving outcomes in patients with subacute pain and exposure to adverse childhood experiences (ACEs) at increased risk of long-term disability.

**Methods:**

Eligible patients were referred by their general practitioner for an open trial of individual case management with group-based education and psychological support sessions and access to allied health services. Measures of pain, disability, and mental health were assessed at baseline, on completion of the 6-month program, and 6 months after completion.

**Results:**

Thirty-nine participants (mean age 51 years, 72% women) completed the program. Pain at baseline was subacute (median duration 9.7 weeks) and of high intensity (median score 8/10), with a mean ACE score of 4.3. After completing the program, participants reported reduced pain severity and interference (~50% reduction), risk of future disability, psychological distress, and number of unhealthy days (~30% reduction) and were all statistically significant (*p* < 0.001). These gains were maintained at 6-months from the beginning of treatment. Higher ACE scores were associated with greater baseline levels of pain interference, risk of future disability, and psychological distress, and with less improvement in pain interference and psychological distress after completing the program.

**Discussion:**

This program suggested pain-related disability and mental health in patients with subacute pain and ACE exposure may be improved, although with reduced efficacy with higher ACE exposure. There need to be further robust investigation to quantify the value of targeted early intervention programs in primary health care settings to help reduce subacute pain persistence and progression to chronic pain in patients at increased risk of long-term disability.

## 1 Introduction

Pain is an aversive physical and emotional state that serves to promote adaptive behaviors to avoid further injury to the body (Becker et al., 2018). It is generally accepted that acute pain may persist during the time of healing, up to 3 months post injury (King, 2013). Subacute pain describes a subset of acute pain that has been present for at least 6 weeks but less than 3 months, although definitions vary (King, 2013). If not managed appropriately, acute and subacute pain may turn into chronic pain (Banerjee and Argaez, 2019). Intervening before pain becomes chronic has significant implications for patients, as well as health and social systems.

People who experience chronic pain can develop an array of stable negative affective and behavioral styles that increase their vulnerability to depression and other mood disorders, and may have a “fixed” or resigned mindset toward pain that limits their active coping strategies (Higgins et al., 2015). However, data suggest that psychosocial treatments for patients with chronic pain that reduce negative affect (or improve positive affect) can help improve pain-related outcomes (Finan and Garland, 2015). People with subacute pain may not have reached the stage where they are resigned to a potential life of pain—remaining focused on “getting better” and open to using different resources (Kellezi et al., 2017). These differences highlight the importance of pain management pathways that deliver timely interventions tailored to the unique needs of patients with acute or subacute pain.

Adverse childhood experiences (ACEs) are potentially traumatic experiences occurring before 18 years of age that can have long-lasting effects on health and well-being and have been shown to associate with maladaptive physiological and behavioral mechanisms (Ranjbar and Erb, 2019). ACEs include events that a child experiences or witnesses (e.g., serious injury/accident, parent divorce, violence, sexual abuse, emotional abuse/neglect), as well as events that undermine a sense of safety, such as growing up in a household with substance abuse, mental health problems, or an incarcerated parent (Groenewald et al., 2020; Holmes et al., 2021). Around 60% of children in Australia have been exposed to at least one ACE, and up to 20% experience three or more ACEs (Holmes et al., 2021). In some cases, exposure to ACEs leads to toxic stress activation (prolonged or permanent abnormal physiological response to stressors), which disrupts normal brain development and increases risk of physical and mental health disorders across the lifespan (Shonkoff et al., 2009; Groenewald et al., 2020). ACEs often co-occur and have a dose-dependent effect, where exposure to multiple ACEs further increases the risk for negative health outcomes, including mental health disorders (Choi et al., 2017; Hughes et al., 2017).

Increasing evidence suggests that ACEs are associated with chronic pain and long-term disability in adults, with higher rates of ACEs in adults with chronic pain than in the general population and double the incidence of chronic pain in adults with ACEs than with no ACEs (Bussieres et al., 2020; Tidmarsh et al., 2022). The role of ACEs on the progression of subacute pain to chronic pain is unclear; however, the association of ACEs with poor mental health may negatively impact mindset toward pain in the subacute phase.

Adults with ACEs who experience subacute pain may be at increased risk for progression to chronic pain, representing a patient population where timely psychosocial intervention could help improve outcomes. We designed a patient-centric pain management program for at-risk adults (defined by presence of ACEs) with subacute pain with the overall goal of reducing or even preventing the persistence of pain and associated disability. This pilot open trial assesses the feasibility and acceptability of the program for improving measures of pain, disability, and mental health from baseline. Additional objectives were to identify any associations between ACEs and baseline pain characteristics and whether ACE scores influenced outcomes of the intervention.

## 2 Materials and methods

### 2.1 Study design

**Figure 1:**
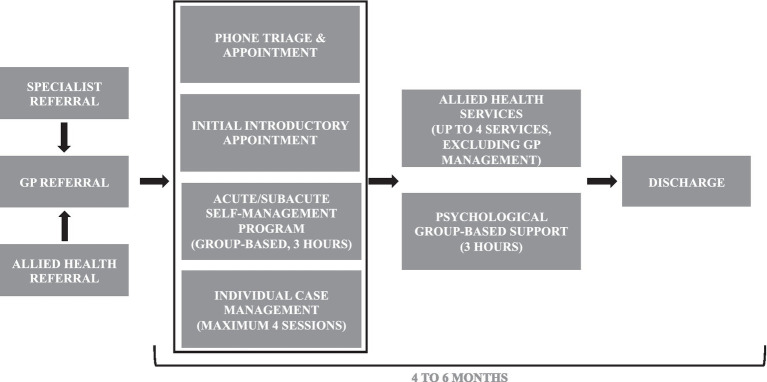
The early intervention acute/subacute self-management pain program pathway.

This was a prospective, observational pilot feasibility trial conducted in Queensland, Australia. Trial enrolment and baseline evaluations began in January 2021, with program completion visits conducted between June and September 2021 and the final 6-month follow-up visit completed in January 2022 (Figure 1).

### 2.2 Participants

Potential participants were identified via their referring general practitioner (GP) after presenting with ongoing pain for 6–12 weeks in the presence of “Yellow Flag” indicators that suggest an increased risk of progression to long-term disability (e.g., unhelpful beliefs about pain and treatment outcomes, fear or anxiety, maladaptive pain coping strategies) (Nicholas et al., 2011). All participants gained entry to the trial via a GP referral form and written informed consent was obtained from all participants involved in the trial. Patients who met the following inclusion criteria were eligible for enrolment: aged 18 years old and above; referred to the program by their GP; experiencing pain for 6–12 weeks; requiring improved self-management strategies and skills to optimize ongoing care; and displaying psychological Yellow Flags. Yellow Flags were used to identify patients at risk of progression to long-term pain and disability because they are more commonly used than ACE scores in the GP setting to evaluate patients with acute and subacute pain. Patients undergoing workers compensation or receiving palliative care, or those with signs of serious pathology (“Red Flags”), suitable for surgical or urgent pain specialist interventions, unable to participate in group education, or give voluntary, informed consent for the ongoing collection of audit data were not eligible to participate. For patients who had undergone surgery in the past 12 weeks, a functional instruction plan was provided with the referral from their GP. A recorded history of ACE was not a prerequisite for trial inclusion.

### 2.3 Outcome measures

Before the initial Pain Service Assessment, participants completed a survey of 25 demographic and pain-related questions (see Supplementary Appendix). To assess the impact of the program on pain outcomes, participants were surveyed at baseline, at the end of the program, and at 6 months after completing the program using existing instruments validated for use in clinical settings. The 10-item self-reported Örebro Musculoskeletal Pain Screening Questionnaire Short Form (ÖMPSQ-Short) assessed the risk of developing long-term workplace disabilities after experiencing pain (Linton et al., 1976). Each item is scored between 0 (lowest risk) and 10 (highest risk) to give an overall measure between 0 and 100, with scores of 50 or more indicating the participant is at higher risk for a future work disability. Pain severity was assessed using the 9-item self-reported Brief Pain Inventory-Short Form (BPI-DF) (Cleeland, 2009). Pain severity was scored by averaging the responses from Q3 to Q6 as a severity score (0 “No pain at all” to 10 “Pain as bad as you can imagine”). Impact of pain on function and independence were scored by averaging the responses from Q9 (consisting of 7 parts) as an interference score (0 “Does not interfere” to 10 “Completely interferes”). Health and wellbeing were assessed using the 14-item Health-Related Quality of Life Scale (HRQoL). “Core healthy days” measured the participants number of unhealthy days over the prior month, including negative physical and mental health, and days where their heath interfered with their regular activities. “Activity limitations” and “healthy days symptoms” measured caring needs, type of pain, and the level of some pain symptoms experienced by the participant. In the current trial, this scale is used to identify the number of unhealthy days per month where a participant is impacted by their condition. Current level of psychological distress was assessed using the 10-item self-reported Kessler Psychological Distress Scale (K10). Each item was scored from 1 (“None of the time”) to 5 (“All of the time”) to give a total K10 score. The overall score was used as a screening measure of psychological distress, ranging from “Low” (10–15) to “Very High” (30+). Participant’s exposure to events or experiences throughout childhood that have been linked to increased mental and physical health problems later in life was assessed using the 10-item self-reported Adverse Childhood Experiences Measure (ACE) scale. The assessment consists of 10 “yes” or “no” questions. Each “yes” answer scored 1 point, with higher total scores indicating more adverse childhood experiences were endured during early life (Felitti et al., 1998). ACE scores demonstrate a “dose–response” relationship with increasingly worse outcomes associated with higher ACE scores, with scores of 4 or more linked to higher rates of a variety of maladaptive lifestyle behaviors, serious health conditions, and mortality (Felitti et al., 1998; Hughes et al., 2017).

### 2.4 Intervention

The Early Intervention Subacute Pain Program was a new, 6-month program operated by PainWise Pty Ltd. in conjunction with the Gold Coast Primary Health Network in Queensland, Australia. The program, designed by a clinical pharmacist, consisted of one 3-h group-based session, four individual case management appointments to help develop a personalized pain management plan, and up to four allied health services (excluding GP management services; Figure 1). Personalized pain management plans include medications, nonpharmacologic therapies (e.g., devices, physical therapy), and lifestyle modifications individually tailored to patient priorities. Allied health services included physiotherapist, exercise physiologist, dietician, psychologist, and pharmacist, and were utilized based on clinical assessment, patient goals, and patient priorities.

A variety of clinicians facilitated the group sessions, including a pharmacist, physiotherapist, exercise physiologist, dietician, and psychologist, which provided patients with education and tools that included presentations, videos, and brochures relevant to pain management (e.g., factors that contribute to ongoing pain, ways to minimize the impact of pain and improve function in everyday life, responsible medication usage). Participants received workbooks to help adapt their personalized pain management plans and develop an action plan in the event of pain flare-ups. At discharge, a pain management plan was provided to participants and their GP.

### 2.5 Statistical analyses

The data were analyzed using IBM SPSS statistics version 28. A missing data analysis revealed an inconsequential proportion (≤5%) of missing data across all key variables of interest (Schafer, 1999; Dong and Peng, 2013). One participant’s responses were manually removed from the data set before analysis due to a failure to complete the post-dataset questionnaire [Participant Identification Number (PID#) 29].

Data were checked for multiple assumptions before performing the analyses. Histogram plots indicated the data was normally distributed for most variables. K10 total scores (pre-intervention) and BPI pain severity (post-intervention and 6-month follow-up) were slightly positively skewed. Skewness (−1.96 to +1.96) and kurtosis (−7 to +7) values for these variables fell within an acceptable range for proceeding with both analysis of variance (ANOVA) and linear regression analysis. While HRQoL number of “unhealthy days” (pre-intervention) indicated platykurtic distribution, analyses proceeded with caution because this variable was a score. The platykurtic distribution was likely caused by the cut-off of 30 days used in the calculation method, impacting the expected scores based on previous literature.

Box-and-whisker plots identified 15 univariate outliers, prompting an examination of the variables and participant responses (PID#s 5, 11, 12, 13, 14, 16, 17, 19, 21, 22, 25, and 33). All outliers were deemed to be within the bounds of realistic answers and were retained in the data set. Homogeneity of variance was measured for each of the ANOVA tests performed, with any violations detailed as relevant. Sphericity was violated in all ANOVA tests, so degrees of freedom were corrected using the Huynh-Feldt adjustment (Tabachnick and Fidell, 2007). The alpha level of significance was set at 0.05 unless otherwise specified.

Correlation analyses were performed to investigate whether participants’ ACE scores were associated with their baseline pain severity, pain interference, risk of future disability, psychological distress, and unhealthy days at pre-test. Spearman’s ρ was used in place of Pearson’s r because unhealthy days failed the test of normality, and all variables failed the test of linearity. Simple regression analyses were performed to determine the extent to which participants’ ACE scores (predictor variable) influenced their outcomes after completing the Early Intervention Subacute Pain Program. The dependent variables used were the mean difference between pre-intervention and post-intervention scores for pain severity, pain interference, risk of future disability, psychological distress, and unhealthy days.

## 3 Results

### 3.1 Participant demographics and baseline pain characteristics

A total of 39 participants with a mean age of 51.3 years completed the program and were included in the analysis (Table 1). Most participants were white (85%) women (72%) who described themselves as casual drinkers (56%, consuming an average of three standard drinks per week) but nonsmokers (56%). 54% of participants were married or living with a partner and 59% had at least one dependent. Twenty five percent of participants reported high school as their highest level of education, with a further 50% attaining trade, technical or vocational training.

**Table 1:** Baseline demographics and pain of participants who enrolled in the early intervention subacute pain program.

Demographic variable	Value (*N* = 39)
Age (years), mean ± SD (range)	51.3 ± 15.9 (24–81)
Sex, n (%)	
Male	11 (28.2%)
Female	28 (71.8%)
Ethnicity, n (%)	
White Non-white	33 (84.6%) 6 (15.4%)
Smokes cigarettes, n (%)	
Yes No	17 (43.6%) 22 (56.4%)
Drinks alcohol (average 3 standard drinks/week), n (%)	
Yes No	22 (56.4%) 17 (43.6%)
Marital status, n (%)	
Single Married *De facto* Separated Divorced Widowed	5 (12.8%) 14 (35.9%) 7 (17.9%) 3 (7.7%) 8 (20.5%) 2 (5.1%)
1 or more dependents, n (%)	23 (59.0%)
Education level, n (%)	
High school Trade/technical/vocational training Associate degree Bachelor’s degree Master’s degree	10 (25.6%) 19 (48.7%) 4 (10.3%) 4 (10.3%) 2 (5.1%)
ACE score, mean ± SD (range)	5.9 ± 3.1 (0–10)
Low ACE score, mean ± SD (range)	2.38 ± 1.04 (0–4)
High ACE score, mean ± SD (range)	7.83 ± 2.01 (5–10)
Duration of pain (weeks), mean ± SD (range)	9.7 ± 2.5 (5–14)
Source of pain, n (%)	
Back or neck problem Fractures, bone, or joint injury Arthritis/rheumatism	20 (51.3%) 14 (35.9%) 5 (12.8%)
Location of pain, n (%)	
Head Upper limb/neck Back Hip/knee Lower limb/feet	3 (7.7%) 14 (35.9%) 15 (38.5%) 10 (25.6%) 2 (2.1%)
Hospitalization for pain in the past 6 months, n (%)	
Yes No	18 (46.2%) 21 (53.8%)
Regular contact with a healthcare professional in the past 6 months, n (%)	
Doctor Pharmacist Physiotherapist Exercise physiologist Dietician Psychologist	39 (100%) 7 (17.9%) 3 (7.7%) 3 (7.7%) 4 (10.2%) 5 (12.8%)
Baseline assessment scores (NRS, 0–10), median (range)	
Pain intensity in the last week Impact of pain on general activity Impact of pain on walking ability Impact of pain on normal work Impact of pain on sleep Impact of pain on mood Impact of pain on enjoyment of life Impact of pain on relationships	8 (6–10) 8 (4–10) 9 (4–10) 8 (5–10) 9 (2–10) 8 (5–10) 7 (4–10) 6 (2–10)

ACE, Adverse Childhood Experiences; NRS, numeric rating scale; SD, standard deviation.

At enrolment, participants completed a baseline survey to describe characteristics of their pain and healthcare (Table 1). Most participants reported mechanical pain in their back, upper limb/neck, or hip/knee regions that had persisted for an average of 9.7 weeks (range 5–14 weeks). While all participants were under regular care from a medical doctor, less than half reported accessing routine multidisciplinary care from allied health professionals. Participants reported a median pain intensity of 8 out of 10 (range 6–10) over the preceding week, with pain causing high levels of interference (median scores of 6–9 out of 10; ranges 2–10) with multiple domains of daily function and quality of life. The mean ACE score of participants was 5.9, standard deviation (SD) 3.1. Among the group, 29.7% had a low ACE score (0–3), and all participants indicated an ACE score of at least 1.

### 3.2 Efficacy of the early intervention subacute pain program

Across the 14 h of the program, 20% of participants time was engaged in psychological interventions, with the remainder physical and educational interventions.

To assess the effectiveness of the program, a series of five one-way repeated measures analysis of variance (ANOVA)s were performed on each of the major survey measures: pain severity (BPI-sf), pain interference (BPI-sf), risk of future disability (ÖMPSQ-Short), psychological distress (K10), and unhealthy days (HRQoL). Overall, these analyses demonstrated significant improvements from baseline (pre-intervention) across all five measures at the end of the program (post-intervention), and that these improvements were generally maintained at the 6-month follow-up visit (Table 2).

**Table 2:** Change from baseline in outcome measures at the end of the early intervention subacute pain program and at 6-month follow-up.

Outcome	Group	Pre-intervention (mean ± SD)	Post-intervention (mean ± SD)	6-Month follow-up (mean ± SD)	ANOVA	Pre-Post difference M_diff_ [95% CI]	Pre-FU difference M_diff_ [95% CI]	Post-FU difference M_diff_ [95% CI]
Pain severity	Overall	6.59 ± 0.71	3.41 ± 0.77	3.49 ± 0.83	*F*(1.09, 39.94) = 517.20* Partial *η*^2^ = 0.933	−3.18* [−3.51, −2.84]	−3.10* [−3.45, −2.75]	0.08^†^ [0.00, 0.16]
	Low ACE	6.71 ± 0.84	3.42 ± 0.81	3.40 ± 0.86		−3.29 [−3.75, −2.83]	−3.23 [−3.60, −2.86]	0.09 [−0.01, 0.18]
	High ACE	6.54 ± 0.64	3.45 ± 0.76	3.63 ± 0.78		−3.08 [−3.44,-2.72]	−2.90 [−3.36, −2.90]	0.07 [−0.02, 0.15]
Pain interference	Overall	7.50 ± 0.75	3.68 ± 0.51	3.66 ± 0.45	*F*(1.10, 40.57) = 1218.79* Partial *η*^2^ = 0.971	−3.81* [−4.09, −3.54]	−3.84* [−4.11, −3.57]	−0.03^‡^ [−0.01, 0.04]
	Low ACE	7.32 ± 0.77	3.68 ± 0.58	3.60 ± 0.36		−3.64 [−4.14, −3.13]	−3.64 [−3.91, −3.37]	−0.04 [−0.13, 0.05]
	High ACE	7.58 ± 0.73	3.71 ± 0.48	3.74 ± 0.57		−3.87 [−4.18, −3.56]	−4.14 [−4.48, −3.80]	0.00
Risk of future disability	Overall	56.82 ± 5.72	38.89 ± 3.90	38.68 ± 3.86	*F*(1.03, 38.06) = 451.19* Partial *η*^2^ = 0.924	−17.92* [−20.07, −15.78]	−18.13* [−20.22, −16.04]	−0.21^‡^ [−0.50, 0.08]
	Low ACE	54.00 ± 5.35	38.15 ± 2.94	38.00 ± 3.52		−15.85 [−18.73, −12.96]	−17.30 [−19.68, −14.93]	−0.17 [−0.48, 0.14]
	High ACE	57.71 ± 5.05	39.46 ± 4.31	39.73 ± 4.23		−18.25 [−19.84, −16.66]	−19.40 [−21.86, −16.94]	−0.27 [−0.66, 0.12]
Psychological distress	Overall	31.24 ± 6.68	21.53 ± 3.85	21.34 ± 3.91	*F*(1.12, 41.414) = 116.75* Partial *η*^2^ = 0.759	−9.71* [−11.96, −7.46]	−9.90* [−12.11, −7.68]	−0.18^‡^ [−0.79, 0.42]
	Low ACE	25.77 ± 3.68	19.92 ± 2.75	20.65 ± 3.92		−5.85 [−7.66, −4.04]	−7.48 [−9.28, −5.68]	−0.35 [−1.17, 0.47]
	High ACE	34.08 ± 6.30	22.88 ± 5.86	22.40 ± 3.78		−11.21 [−14.21, −8.20]	−13.60 [−16.48, −10.72]	0.07 [−0.08, 0.21]
Unhealthy days^§^	Overall	29.08 ± 2.48	21.13 ± 4.39	20.95 ± 6.49	*F* (1.06, 39.04) = 63.98* Partial *η*^2^ = 0.634	−7.95* [−10.42, −5.48]	−8.13* [−10.66, −5.60]	−0.18^‡^ [−0.65, 0.28]
	Low ACE	28.31 ± 3.33	23.08 ± 6.08	20.30 ± 7.23		−5.23 [−8.57, −1.89]	−8.35 [−11.35, −5.35]	0.00
	High ACE	29.46 ± 1.89	20.29 ± 5.74	21.93 ± 5.23		−9.17 [−11.46, −6.87]	−7.80 [−10.63, −4.97]	−0.47 [−1.47, 0.53]

*p < 0.001; ^†^*p* = 0.049; ^‡^Not significant, *p* > 0.05. ^§^Interpret with caution, as data violated assumption of homogeneity of variance.

Pain severity was reduced by 38.9% and pain interference was reduced by approximately 42.2% at the end of the intervention. Risk of future disability was reduced by 17.9%, psychological distress was reduced by 24.3%, and unhealthy days were all reduced by approximately 34.7% at the end of the intervention. These were all statistically significant compared to baseline (*p* < 0.001, Table 2).

### 3.3 Association of ACE scores with baseline pain characteristics

For the participants with data available (*n* = 24), ACE scores were significantly, positively correlated with pain interference [*ρ*(38) = 0.32, *p* = 0.050], risk of future disability [*ρ*(38) = 0.39, *p* = 0.016], and psychological distress [*ρ*(38) = 0.56, *p* < 0.001]. This suggests that people with subacute pain who experience more ACEs are more likely to present with higher levels of pain interference, risk of future disability, and psychological distress. ACE scores were not associated with baseline pain severity [*ρ*(38) = 0.11, *p* = 0.511] and unhealthy days per month [*ρ*(38) = 0.14, *p* = 0.406].

### 3.4 Ability of ACE scores to predict positive outcomes after participation in the early intervention subacute pain program

Regression analyses showed that ACE scores significantly influenced participants’ reduction in pain interference after completing the program [*F*(1, 36) = 4.35, *p* = 0.044, R2 = 0.11, adjusted R2 = 0.08], accounting for 11% in the variance of pain interference from pre-intervention to post-intervention. Higher ACE scores were associated with a smaller effect size (*β* = − 0.33, *p* = 0.044), indicating that the program was less beneficial for reducing pain interference in those with higher ACE scores than in those with lower ACE scores.

ACE scores also significantly influenced participants’ reduction in psychological distress after completing the program [*F*(1, 36) = 28.35, *p* < 0.001, R2 = 0.44, adjusted R2 = 0.43], accounting for 44% in the variance of psychological distress from pre-intervention to post-intervention. Higher ACE scores were associated with a smaller effect size (*β* = −0.66, *p* < 0.001), indicating that the program was less beneficial for reducing psychological distress in those with higher ACE scores than in those with lower ACE scores.

ACE scores did not predict the pre-intervention to post-intervention reduction in pain severity [*F*(1, 36) = 0.155, *p* = 0.696, R2 = 0.00, adjusted R2 = −0.02], risk of future disability [*F*(1, 36) = 1.11, *p* = 0.298, R2 = 0.03, adjusted R2 = 0.00], or unhealthy days [*F*(1, 36) = 0.92, *p* = 0.344, R2 = 0.03, adjusted R2 = 0.00].

## 4 Discussion

Exposure to ACEs has previously been associated with negative health outcomes and maladaptive behaviors and may increase risk for developing chronic pain and disability (Hughes et al., 2017; Bussieres et al., 2020; Groenewald et al., 2020; Tidmarsh et al., 2022). In our holistic early intervention subacute pain program participants with higher ACE exposure derived less pain and functional benefit than those with lower ACE exposure. However, our feasibility pilot open trial suggests that delivery of early psychosocial education and support to at-risk patients experiencing subacute pain may still be beneficial to help prevent progression to chronic pain, filling an identified gap in primary health services for pain management in Australia (De Morgan et al., 2022).

To our knowledge, this is the first-time group-based self-management education and psychological support has been studied in combination with personalized case management and allied health services with the aim to reduce pain persistence and the risk of long-term disability. The delivery of both psychological and physical therapy together in one program may be necessary to derive benefit. Previously, a systematic review and meta-analysis across 11 clinical trials found psychological therapy may impact disability and coping with pain, but has not definitively shown a significant effect on pain intensity (Berube et al., 2021). In our program, the participants presented with high-intensity mechanical pain that caused substantial interference to their normal activities for a median duration of 10 weeks and were at increased risk of progression to long-term distress and disability. After completing the program, the participants recorded reductions in pain severity, pain interference and had a reduction in risk of future disability, psychological distress, and number of unhealthy days per month at the end of the program, compared to baseline. These improvements were maintained at the 6-month follow-up visit. However, with the current feasibility pilot open trial design, it is not possible to rule out that reductions in pain and improvement in functioning were not due to other factors. Without a control group, it is not clear if the intervention itself was associated with a change in pain severity or the contribution of other effects, including the natural course of healing, regression to the mean or even the placebo effect. It may suggest a randomized controlled trial would be warranted to quantify the effectiveness of the program in meeting its aim. In addition, the heterogeneous trial population and small number of participants limited the ability to assess the effect of different potential confounding factors (e.g., access to routine care, smoking, alcohol, education) on the outcomes of the intervention. The trial was also limited by the use of patient-reported data based on questionnaires and surveys, which may reflect recall bias.

We observed that higher ACE scores predicted greater risk of long-term disability due to pain persistence, as well as greater impact of pain on mental health. Similar associations between ACE exposure and negative physical and psychological outcomes have been previously described for patients with chronic pain (Tidmarsh et al., 2022). While the benefit of the Early Subacute Pain Intervention Program for reducing pain interference and psychological distress declined as the number of ACEs increased, in our small patient cohort, ACE scores alone did not predict changes in pain severity, risk of future disability, or number of unhealthy days. It is unknown whether the overall benefit from this intervention is truly not impaired across all domains by higher levels of exposure to ACEs, or whether this is a limitation of sample size. However, treatment response and improvement irrespective of number of ACEs has also been reported previously in patients with chronic pain participating in a 10-week multidisciplinary pain rehabilitation program (Craner and Lake, 2021).

Interestingly, our cohort had a high proportion (43.6%) of smokers compared to the Queensland adult general population (10.4%) (Queensland Government, 2023). However, smoking was not found to correlate with ACE scores, baseline pain severity, pain or functional outcomes (data not shown). Future studies should continue to monitor smoking status effect in larger sample sizes and duration of follow up.

Overall, this feasibility pilot open trial suggests further studies are warranted to understand the value of biopsychosocial self-management programs specifically targeted at adults with subacute pain at increased risk of progression to chronic pain for improving pain-related disability and mental health. It highlights the importance of identifying at-risk patients and delivering early interventions in the primary health care setting to help alleviate the need for long-term and chronic pain management programs. Future work is needed to evaluate the efficacy of this intervention relative to a randomized control group who receive standard of care and a more homogeneous or strictly defined cohort that could be stratified based on key confounding factors, including presence or absence of ACEs.

## Data availability statement

The raw data supporting the conclusions of this article will be made available by the authors, without undue reservation.

## Ethics statement

The studies involving humans were approved by the Bond University Research Human Ethics Committee. The studies were conducted in accordance with the local legislation and institutional requirements. The participants provided their written informed consent to participate in this study.

## Author contributions

JM: Conceptualization, Investigation, Methodology, Writing – original draft. PS: Conceptualization, Formal analysis, Methodology, Writing – original draft, Writing – review & editing. CP: Conceptualization, Writing – original draft, Writing – review & editing.
