# Supplementary material for: Higher adverse childhood experiences interference with targeted early intervention to reduce persistence of adult subacute pain: a feasibility open trial
Source: Front Psychol. 2023 Nov 23;14:1270598. doi: 10.3389/fpsyg.2023.1270598 (PMC10702217; doi:10.3389/fpsyg.2023.1270598)

**Higher adverse childhood experiences interference with targeted early intervention to reduce persistence of adult subacute pain: a feasibility open trial**

**Supplementary Appendix**


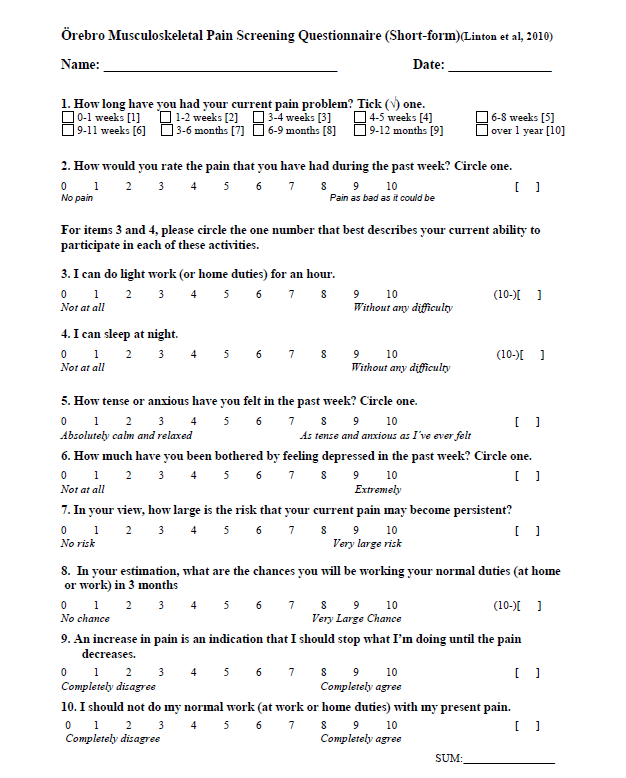


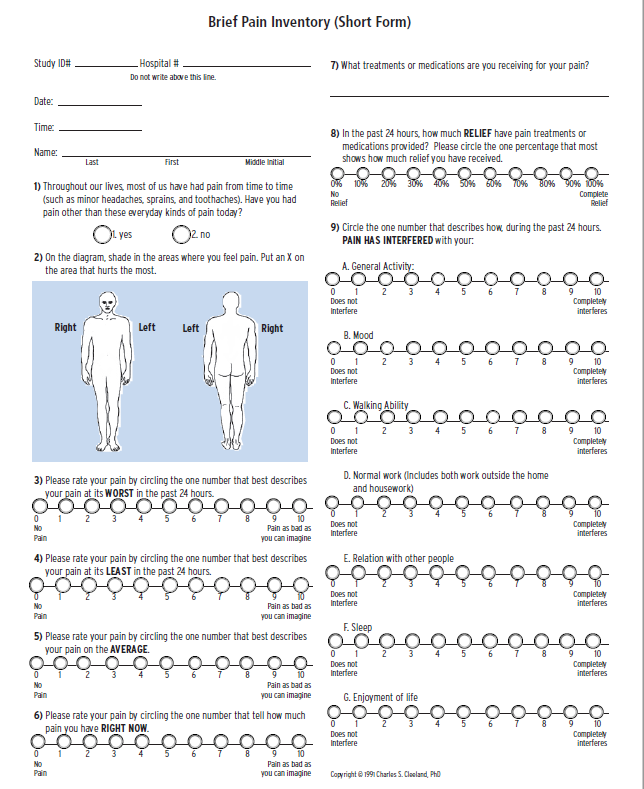


**Health-Related Quality of Life Scale**

*Core Healthy Days Module*

1. Would you say that in general your health is:

1 = Excellent
2 = Very good
3 = Good
4 = Fair
5 = Poor

2. Now thinking about your physical health, which includes physical illness and injury, for how many days during the past 30 days was your physical health not good?

1 = number of day (fill in the blank); 2 = none.

3. Now thinking about your mental health, which includes stress, depression, and problems with emotions, for how many days during the past 30 days was your mental health not good?

1 = number of day (fill in the blank); 2 = none.

If you answered “none” to questions 2 and 3, skip question 4 below:

4. During the past 30 days, for about how many days did poor physical or mental health keep you from doing your usual activities, such as self-care, work, or recreation?

1 = number of day (fill in the blank); 2 = none.

*Activity Limitations Module*

Instructions: These next questions are about physical, mental, or emotional problems or limitations you may have in your daily life.

5. Are you LIMITED in any way in any activities because of any impairment or health problem?

If the answer is no, skip to “Healthy Days Symptoms Module.”

1 = yes; 2 = no.

6. What is the MAJOR impairment or health problem that limits your activities?

1 = arthritis/rheumatism; 2 = back or neck problem; 3 = fractures, bone/joint injury; 4 = walking problem; 5 = lung/breathing problem; 6 = hearing problem; 7 = eye/vision problem; 8 = heart problem; 9 = stroke problem; 10 = hypertension/high blood pressure; 11 = diabetes; 12 = cancer; 13 = depression/anxiety/emotional problem; 14 = other impairment/problem.

7. For HOW LONG have your activities been limited because of your major impairment or health problem?

Fill in the blank ……

8. Because of any impairment or health problem, do you need the help of other persons with your PERSONAL CARE needs, such as eating, bathing, dressing, or getting around the house?

1 = yes; 2 = no.

9. Because of any impairment or health problem, do you need the help of other persons in handling your ROUTINE needs, such as everyday household chores, doing necessary business, shopping, or getting around for other purposes?

1 = yes; 2 = no.

*Healthy Days Symptoms Module*

10. During the past 30 days, for about how many days did PAIN make it hard for you to do your usual activities, such as self-care, work, or recreation?

11. During the past 30 days, for about how many days have you felt SAD, BLUE, or DEPRESSED?

12. During the past 30 days, for about how many days have you felt WORRIED, TENSE, or ANXIOUS?

13. During the past 30 days, for about how many days have you felt you did NOT get ENOUGH REST or SLEEP?

14. During the past 30 days, for about how many days have you felt VERY HEALTHY AND FULL OF ENERGY?

NOTE:

To calculate the unhealthy days score for each participant, sum the number of physically unhealthy and mentally unhealthy days. The maximum score is 30 unhealthy days, even if the number of unhealthy days totals more than 30. To calculate a healthy day’s score, subtract the number of unhealthy days from 30.

**Adverse Childhood Experiences Measure**

**Please circle Yes or No for the following questions.**

**While you were growing up, during your first 18 years of life:**

| 1. Did a parent or other adult in the household often …  Swear at you, insult you, put you down, or humiliate you?  or  Act in a way that made you afraid that you might be physically hurt. | YES | NO |
| --- | --- | --- |
| 2. Did a parent or other adult in the household often …  Push, grab, slap, or throw something at you?  or  Ever hit you so hard that you had marks or were injured? | YES | NO |
| 3. Did an adult or person at least 5 years older than you ever…  Touch or fondle you or have you touch their body in a sexual way?  or  Try to or actually have oral, anal, or vaginal sex with you? | YES | NO |
| 4. Did you often feel that …  No one in your family loved you or thought you were important or special?  or  Your family didn’t look out for each other, feel close to each other, or support each other? | YES | NO |
| 5. Did you often feel that …  You didn’t have enough to eat, had to wear dirty clothes, and had no one to protect you?  or  Your parents were too drunk or high to take care of you or take you to the doctor if you needed it? | YES | NO |
| 6. Were your parents ever separated or divorced? | YES | NO |
| 7. Was your mother or stepmother:  Often pushed, grabbed, slapped, or had something thrown at her?  or  Sometimes or often kicked, bitten, hit with a fist, or hit with something hard?  or  Ever repeatedly hit over at least a few minutes or threatened with a gun or knife? | YES | NO |
| 8. Did you live with anyone who was a problem drinker or alcoholic or who used street drugs? | YES | NO |
| 9. Was a household member depressed or mentally ill or did a household member attempt suicide? | YES | NO |
| 10. Did a household member go to prison? | YES | NO |

**Kessler Psychological Distress Scale**


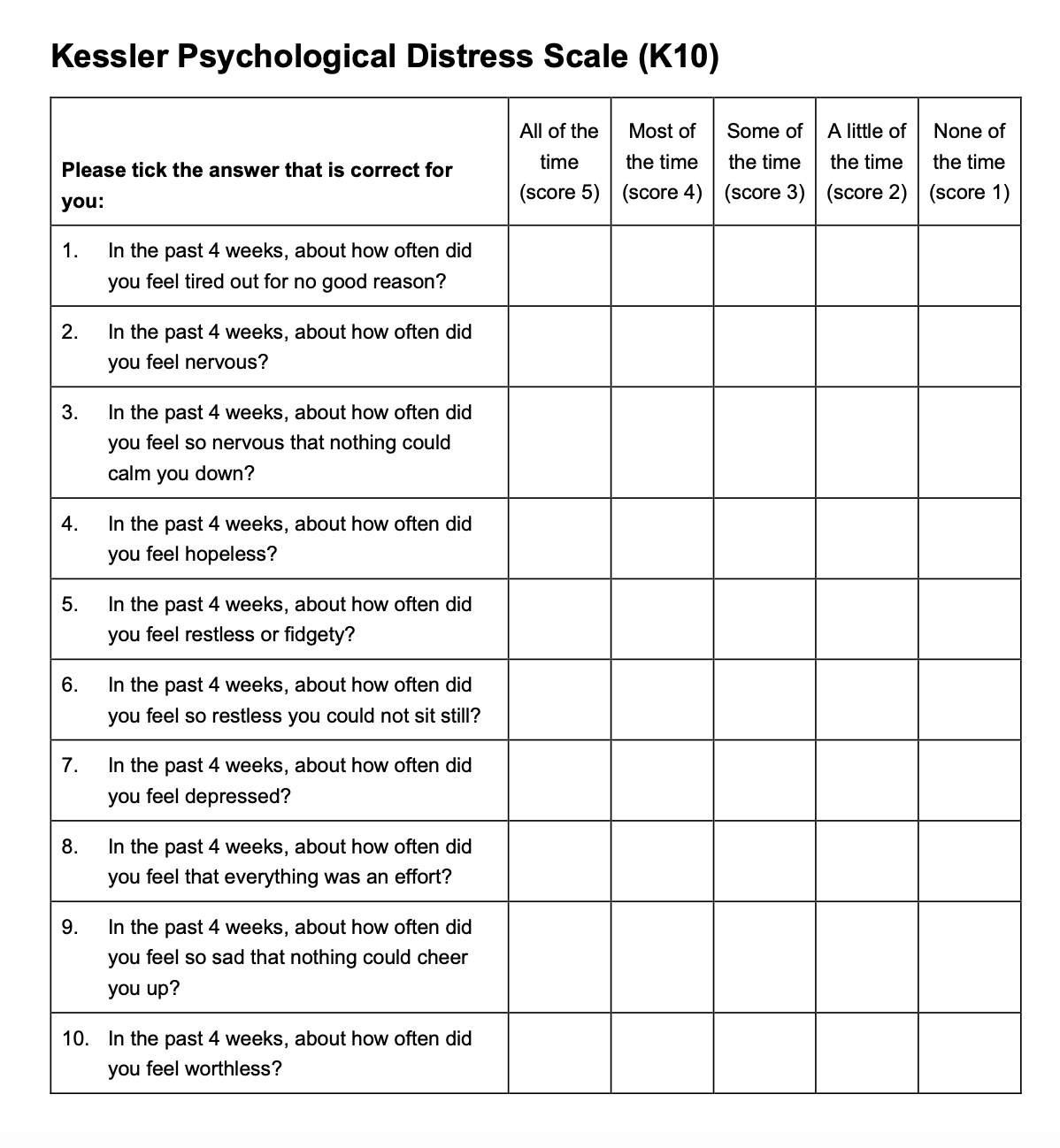

Supplement: Supplementary file 1 [file Data_Sheet_1.docx]
